# Patterns of housework performance in the United States before, during, and after the Great Recession

**DOI:** 10.3389/fsoc.2023.1153321

**Published:** 2023-09-22

**Authors:** Shannon N. Davis

**Affiliations:** Office of Faculty and Academic Affairs, George Mason University Korea, Incheon, Republic of Korea

**Keywords:** Great Recession, housework, domestic labor, household chores, LPA

## Abstract

Housework is a key area of research across many academic fields as it represents the intersection of micro- and macro-level gender dynamics. Despite many shifts in both women's and men's economic activities, and men's changing gender beliefs, women remain largely responsible for the management and performance of domestic labor. Given the relationship between paid employment and household work, this research describes patterns of women's and men's housework before, during, and after the Great Recession. Using American Time Use Survey data, I perform latent profile analysis to document the distributions of housework tasks and time for women and men across these three time periods. While women perform the majority of housework across the time frame, women and men converge in their time during the Recession. Further, men's time becomes more varied and more similar to women's Post-Recession. The findings in this research brief highlight the connections between macro-level change and micro-level behavior.

## Introduction

Housework is a key area of research across many academic fields as it represents the intersection of micro- and macro-level gender dynamics (see Scarborough and Risman, 2017, for a discussion of these intersections). Despite many shifts in both women's and men's economic activities, and men's changing gender beliefs, women remain largely responsible for the management and performance of domestic labor (Bianchi et al., 2012). Further, there are patterns to the tasks that women and men perform when engaging in housework (e.g., Arrighi and Maume, 2000; Kolpashnikova and Kan, 2021).

While scholars studying domestic labor have found consistently that time in the labor market has a negative relationship with housework (e.g., Besen-Cassino and Cassino, 2014), the recent Great Recession provided a massive economic shock that upended employment opportunities for women and men who desired to be in the labor market. The Great Recession, as coined by economists, officially began in December 2007 and ended in 2011 (Boushey, 2011). This Recession was the longest and deepest since World War II, a global economic event that touched all segments of the labor market in some way (Goodman and Mance, 2011; Kochhar, 2011). Berik and Kongar (2013) documented the effects of the Great Recession and extended Recession after the jobless recovery on married mother's and fathers' time allocation across paid work, unpaid work (housework, child care, and adult care), personal care, and leisure. They found, among other things, that the Recession did not reduce the gender disparity in the performance of housework (Berik and Kongar, 2013). This is consistent with other findings, where men's time on housework relative to women's was both less variable and more responsive to external forces (Khitarishvili and Kim, 2014), even as women performed less housework and men performed more after the Recession relative to the few years prior to the Recession (Davis and Greenstein, 2020).

This paper expands research on housework performed in the early part of the 21st century in the United States by documenting patterns in housework task allocation among women and men in three distinct time periods: before, during, and after the Great Recession. This exploratory analysis provides insights into how average American women and men may or may not have shifted their household labor time during and after the Recession. The exploratory comparison of patterns across the three time periods allows examination of possible associations of gendered task allocation and external social forces. For example, the patterns of task allocation between and among women and men may have become more similar to one another due to the pressures of the Great Recession, consistent with theories of time availability (see Gough and Killewald, 2011, for a review). Alternatively, the perceived gendered nature of the early Recession (Christensen, 2015) may have led to both women and men engaging in gender deviance neutralization (Greenstein, 2000; but see Gough and Killewald, 2010; Hook, 2017), wherein women perform more housework and men perform less when their economic circumstances may threaten their gendered identities. In this research brief, I provide insights into the basic patterns of women's and men's task allocation across the three time periods but do not specifically test hypotheses due to the exploratory nature of the analysis.

## Method

### Data

These analyses utilize data gathered for the American Time Use Survey (ATUS), pooling 13 cross-sections (2003–2015) using the ATUS extract builder (Hofferth et al., 2013) that contained a total of 170,842 respondents. The ATUS is a nationally representative sample sponsored by the Bureau of Labor Statistics focusing on how Americans spend their time. Respondents are drawn from participants in the Current Population Survey (CPS), which samples the civilian noninstitutional population. Each state is sampled proportional to its share of the national population. Hispanics, non-Hispanic Blacks, and households with children are oversampled. One adult in each sampled household is selected for interview. Respondents are interviewed by telephone about their time use starting at 4 a.m. the previous day and continuing for 24 hours. For more details about the ATUS sampling and interview procedures, see Bureau of the Census. (2019).

From the 170,842 ATUS respondents across the 13 cross-sections I analyzed reports of housework tasks from respondents who were currently in a marital or nonmarital union. Of the 170,842 respondents, 79,875 were not currently in a marital or nonmarital union (for reference, 6,950 respondents (7.6%) were in a nonmarital union, while 519 (about.6%) were in same-sex couples). The 91,057 respondents who were currently in a union were classified based upon the time period in which they responded to the survey: Pre-Recession (2003-2007), Recession and jobless recovery (2008–2010), and Post-Recession (2011–2015).

In this paper, I examine household labor, operationalized as the total number of minutes per day spent on household tasks. These tasks were part of the time diary, where respondents were asked about the activities performed during the day as well as the duration of time spent on each activity. I organized the ATUS housework categories into eight general task groupings, five of which are considered what has been called “routine housework” by others using these data (Hook, 2017; Ruppanner et al., 2021): Meals, Kitchen Work, Cleaning, Grocery Shopping, Laundry. I also included tasks that are considered by scholars as unpaid household labor but are less routine (Krantz-Kent, 2009): Bills, Driving, and Yardwork.

### Analytic approach

I performed Latent Profile Analysis (LPA) in Mplus (Muthén and Muthén, 1998–2017), an approach that seeks to uncover patterns of behaviors in the data. These patterns are categorized as classes. As an analytic approach, LPA is designed to examine large amounts of continuous data for patterns that represent classes of people in the population. It is appropriate for these data as it reveals distinct groups from among the data when membership markers for groups are unknown. I performed LPA separately for women and men within each of the three time periods. I utilized multiple fit statistics to evaluate the models and determined the class enumeration in each time period for women and men as described in Tables 1, 2 (see the Appendix for the fit statistics). Table 1 presents women's distributions of time within and across the three time periods, while Table 2 presents the same distributions for men. The classes are presented in ascending order by minutes spent on housework. The naming conventions/labels used for the classes are purely descriptive and are of my own creation, used to enable further comparisons among the patterns documented. I provide further explanation of the labels I attributed to the classes in the Results section.

**Table 1:** Description of women's patterns of housework across three time periods.

**Class label**	**Total housework minutes/day**	**Prevalence**
**Pre-Recession (*****N*** = **21,404)**	**Avg: 148.29**	
Low housework traditional	104.58	0.632
Average but managerial	178.02	0.073
Mostly average	179.23	0.019
Above average some masculine	213.67	0.014
High housework neutral masculine	224.50	0.063
High housework neutral–feminine	224.56	0.006
High housework mostly feminine	234.14	0.111
High housework traditional	251.34	0.082
**Recession (*****N*** = **10,683)**	**Avg: 143.30**	
Low housework traditional	121.58	0.789
Average	191.10	0.023
High housework with masculine	229.25	0.055
High housework traditional	231.89	0.132
**Post-Recession (*****N*** = **15,635)**	**Avg: 142.03**	
Low housework traditional	112.96	0.729
Average on most	171.84	0.008
Mostly average with yardwork	174.04	0.008
Mostly average with driving	177.36	0.081
Above average some masculine	194.68	0.012
High housework neutral–masculine	205.01	0.006
High housework all feminine	216.74	0.055
High housework traditional	259.43	0.102

**Table 2:** Description of men's patterns of housework across three time periods.

**Class label**	**Total housework minutes/day**	**Prevalence**
**Pre-Recession (*****N*** = **19,178)**	**Avg: 62.35**	
Low housework traditional	42.43	0.784
Transitional	95.88	0.070
Transitional managerial	95.88	0.007
Non-traditional	111.45	0.005
Non-traditional more feminine	122.10	0.045
Trad housework plus yard work	175.88	0.088
**Recession (*****N*** = **9,658)**	**Avg: 63.98**	
Low housework traditional	44.30	0.783
Transitional managerial	97.26	0.075
Transitional	98.87	0.012
Non-traditional	120.49	0.044
Traditional housework plus yard work	179.47	0.086
**Post-Recession (*****N*** = **14,499)**	**Avg: 66.56**	
Low housework traditional	38.61	0.715
Traditional plus bills	91.11	0.067
Transitional	95.68	0.009
Non-traditional	112.16	0.007
Transitional managerial	125.23	0.049
Transitional plus groceries	145.04	0.070
Traditional housework plus yard work	180.28	0.083

## Results

To address the questions regarding changing patterns of housework for women and men, I performed LPA separately by sex. I present the separate analyses here, followed by a description of the comparison of women and men in the Discussion section.

### Women

**Figure 1:**
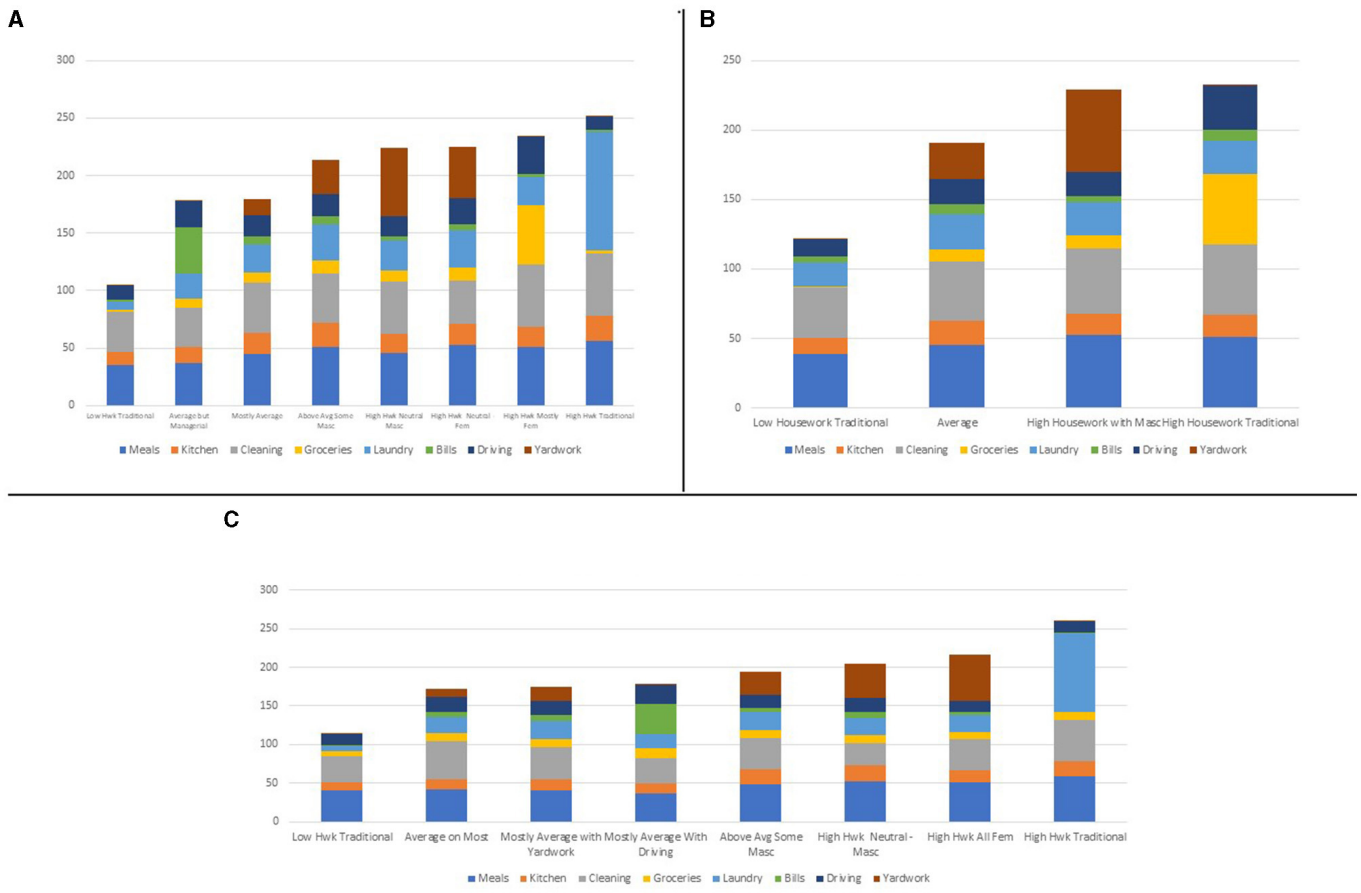
Women's housework time by task across three time periods. **(A)** Women's housework time in minutes: Pre-Recession. **(B)** Women's housework time in minutes: Recession. **(C)** Women's housework time in minutes: Post-Recession.

While Table 1 provides an overview of women's housework patterns, Figure 1 depicts women's patterns of housework by task across the three time periods. Women had eight distinct patterns (classes) Pre- and Post-Recession (Panels A and C) while only four patterns (classes) during the Recession (Panel B). As noted in Table 1 and Panel A, the most prevalent pattern of housework was a traditional division, where women performed only routine tasks, albeit spending the least amount of time across all groups. The other seven classes of women during the Pre-Recession period performed substantially more housework than did the most prevalent group (the group I call Low Housework Traditional). The two groups who performed slightly more than the Low Housework Traditional group performed more than one hour more per week of tasks, mostly focused on average amounts of routine work but adding in the managerial tasks or yardwork. All remaining groups during the Pre-Recession period spent approximately four hours or more during the day on housework. These high housework groups varied in the combination of tasks performed, although the High Housework Traditional class spent a lot of time on routine tasks, more time on housework than the Low Housework Traditional class did in total.

Women on average spent five fewer minutes on housework during the Recession than did women before the Recession. Women's work became less varied during the Recession, perhaps as a response to the external economic changes and constrained opportunities. Not only does Table 1, Figure 1B show that there were only four classes, but the difference between the time spent by those who spent the least (Low Housework Traditional) and most (High Housework Traditional) time on housework was smaller than that same difference Pre-Recession. This is because the Low Housework Traditional women spent about 15 more minutes per day than did their counterparts Pre-Recession and the High Housework Traditional women spent about 20 fewer minutes per day than their Pre-Recession counterparts. Overall, the patterns of the women's task distribution during the Recession reflect two kinds of women: women who focused almost exclusively on the home (Low Housework Traditional) and women who, to differing degrees, engaged with activities outside of the home (the other three classes), as noted by the time spent on grocery shopping, driving, and yardwork that differentiates the Low Housework Traditional class from the other three classes of women during the Recession.

Post-Recession women spent approximately the same amount of time per day on housework as did their counterparts during the recession and six fewer minutes per day than did their Pre-Recession counterparts, as noted in Table 1, Figure 1C. In many ways, however, Post-Recession women were quite similar to Pre-Recession women. The number of distinct patterns of housework tasks increased to Pre-Recession levels, with eight patterns emerging from the data. Interestingly, the Low Housework Traditional class increased in prevalence relative to the Pre-Recession time period and increased in the minutes per day spent on housework. There are only three high housework classes that emerged during the Post-Recession rather than four, with more women clustered around three rather than almost four hours of housework per day. In addition, the High Housework Traditional class performed ~8 min more housework per day and were a larger percentage of the overall group of women, than were their counterparts during the Pre-Recession time period.

### Men

**Figure 2:**
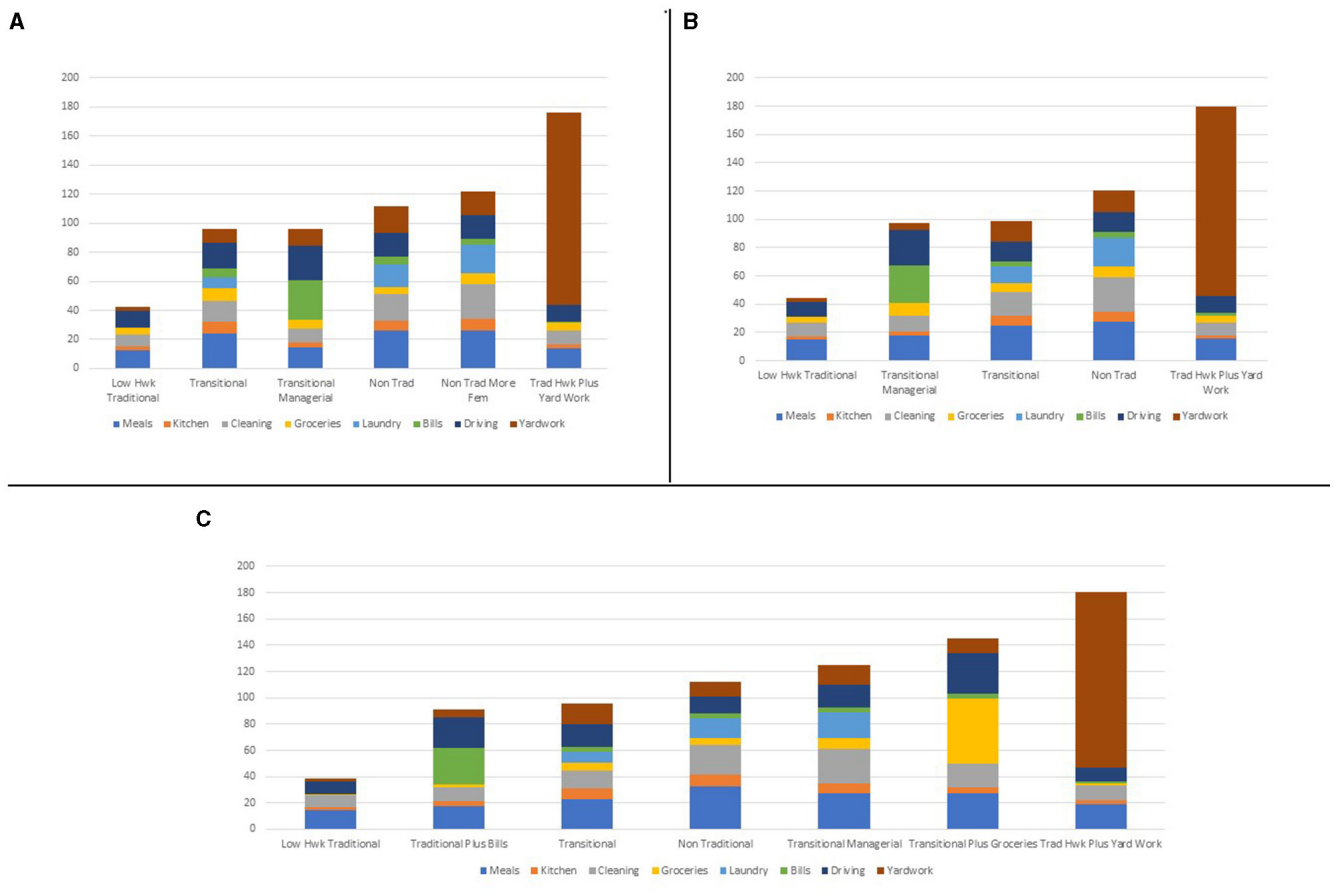
Men's housework time by task across three time periods. **(A)** Men's housework time in minutes: Pre-Recession. **(B)** Men's housework time in minutes: Recession. **(C)** Men's housework time in minutes: Post-Recession.

Table 2 provides an overview of men's housework patterns, while Figure 2 depicts men's patterns of housework across the three time periods. Men had six distinct patterns (classes) Pre-Recession (Panel A), five patterns during the Recession (Panel B), and seven patterns after the Recession (Panel C). Traditional and less traditional distributions were seen in all three time periods, with one interesting group with a traditional pattern and high levels of yard work in each time period. However, the most prevalent group across all three time periods were the Low Housework Traditional men. These Low Housework Traditional men were distinctly different from the other men in each time period. Further, within each time period, the men who were not engaged in traditional distribution of time were quite similar to one another overall. As noted above, the Traditional Housework plus Yard Work group is distinct in each time period, with this group only distinguishing themselves from the Low Housework Traditional men by the immense amount of yardwork per day that they performed.

Also within each time period is a set of classes I call Transitional, named after the transitional group found in Hochschild's (1989) classic monograph on the division of housework. These men perform mostly masculine tasks, but each class adds in some feminine task(s) that distinguishes them from the Low Housework Traditional men. For example, during the Pre-Recession time period, the Transitional men spend more time per day on all of the routine tasks than do their Low Housework Traditional counterparts. During the Recession, Transitional Managerial men performed more “managerial” tasks than did their Low Housework Traditional counterparts, specifically in that they spent more time paying bills and driving. During the Post-Recession time period, the Transitional plus Groceries class performed a substantial amount of time per day on grocery shopping relative to their Low Housework Traditional counterparts.

Unlike women, men performed slightly more housework per day after the Recession than before, although women perform 2.5 times as much housework per day across each time period on average. Post-recession, the Low Housework Traditional men performed less housework per day than did their Pre-Recession counterparts. However, the Low Housework Traditional men performed more minutes per day than did either those in the Pre- (+2 min) or Post-Recession (+6 min) time periods.

## Discussion and conclusion

Housework is a task performed in all households but is mostly studied as a gendered behavior in multi-person residences. This exploratory analysis examined patterns of housework time among married women, among married men, and between married (though not to each other) women and men in three time periods (before, during, and after the Great Recession).

Using American Time Use Survey data, I examined the extent to which the Great Recession changed patterns in women's and men's housework performance. There was a difference in the distribution of tasks during the Great Recession for both women and men. There were fewer distinct patterns of task distribution for both women and men during the Great Recession than before and after. I suggest that the economic shock placed on households during the Recession may have created more within-sex similarity as women and men structured their home life in response to the constrained opportunities in the public sphere, an explanation consistent with time availability approaches.

Women's time distribution before and after the Recession was more similar than men's time distribution, suggesting that the Great Recession likely had more of a social change effect on men than on women. After the economic shock, women's patterns were quite similar to the Pre-Recession time period. Men's patterns reflected more variability, more feminine tasks, and more distinct groups. However, one point did not change (low housework). Traditional men, men engaging in only traditionally masculine tasks, were the most prevalent and distinctly different from other men across all three time periods. While the Great Recession may have led some men to become less traditional, the path of least resistance remains a traditionally masculine performance of very little housework.

Besen-Cassino (2019) argues that the Great Recession likely affected men's housework participation because their economic insecurity was perceived as a threat to their masculinity. This research found that men's housework became less traditionally gendered, on average, after the Great Recession. Kidane (2019) found that immigrant Hispanic men performed more housework than did other men during and after the Recession, suggesting a specifically racialized interpretation of masculinity that may be threatened by the economic uncertainty that surrounded the Recession. This is an important vein of inquiry that will need to be pursued. Additional lines of inquiry include demographic correlates of the patterns within each time period that extend beyond race/ethnicity. In addition, physical task performance is not the only measure of housework (e.g., the cognitive dimension—see Daminger, 2019, for more details). Other scholars may seek to uncover whether a composite of all components of housework follow the same patterns documented herein that were found before, during, and after the Great Recession.

The contribution of this article is to uncover, through a unique descriptive analysis, how gendered patterns of housework tasks themselves shift in response to the Great Recession. Given the attention being paid to gendered housework performance during the COVID-19 pandemic, it is encouraged that future authors evaluate the extent to which patterns of housework tasks also shifted as a result of remote work and job loss during the pandemic. The intersection of economic disruption and cultural norms that occurred within the United States in the Great Recession suggest that it will take something like a global pandemic to move the needle on prevalent patterns of the performance of housework that disproportionately burden women with routine tasks.

## Data availability statement

Publicly available datasets were analyzed in this study. This data can be found here: https://www.bls.gov/tus/data.htm.

## Ethics statement

Ethical approval was not required for the study involving humans in accordance with the local legislation and institutional requirements. Written informed consent to participate in this study was not required from the participants or the participants' legal guardians/next of kin in accordance with the national legislation and the institutional requirements.

## Author contributions

The author confirms being the sole contributor of this work and has approved it for publication.
