# Supplementary material for: Patterns of housework performance in the United States before, during, and after the Great Recession
Source: Front Sociol. 2023 Sep 22;8:1153321. doi: 10.3389/fsoc.2023.1153321 (PMC10556670; doi:10.3389/fsoc.2023.1153321)
Supplement: Supplementary file 1 [file Table_1.docx]

**APPENDIX**

| **Table A1. Model Fit Statistics Used to Determine Class Enumeration for Female Respondents** | | | | | | | | |
| --- | --- | --- | --- | --- | --- | --- | --- | --- |
|  | **Classes** | **# Parameters** | **AIC** | **BIC** | **BLRT *p* value** | **VLMR *p* value** | **LMRT *p* value** | **Entropy** |
| Pre-Recession | 1 | 16 | 1577714.530 | 1577842.071 | - | - | - | - |
|  | 2 | 25 | 1531090.206 | 1531289.490 | 0.000 | 0.000 | 0.000 | 1.000 |
|  | 3 | 34 | 1501358.890 | 1501629.916 | 0.000 | 0.000 | 0.000 | 1.000 |
|  | 4 | 43 | 1478830.165 | 1479172.932 | 0.000 | 0.000 | 0.000 | 0.998 |
|  | 5 | 52 | 1463521.111 | 1463935.620 | 0.000 | 0.004 | 0.005 | 0.998 |
|  | 6 | 61 | 1446443.769 | 1446930.020 | 0.000 | 0.000 | 0.000 | 0.998 |
|  | 7 | 70 | 1431583.025 | 1432141.019 | 0.000 | 0.000 | 0.000 | 0.987 |
|  | 8 | 79 | 1425018.766 | 1425648.501 | 0.000 | 0.000 | 0.000 | 0.973 |
|  | 9 | 88 | 1416170.221 | 1416871.698 | 0.000 | 0.077 | 0.078 | 0.988 |
|  |  |  |  |  |  |  |  |  |
| Recession | 1 | 16 | 783432.844 | 783549.266 | - | - | - | - |
|  | 2 | 25 | 760287.242 | 760469.152 | 0.000 | 0.000 | 0.000 | 1.000 |
|  | 3 | 34 | 745579.774 | 745827.172 | 0.000 | 0.000 | 0.000 | 1.000 |
|  | 4 | 43 | 734278.372 | 734591.258 | 0.000 | 0.000 | 0.000 | 0.990 |
|  | 5 | 52 | 724998.223 | 725376.596 | 0.000 | 0.070 | 0.071 | 0.991 |
|  |  |  |  |  |  |  |  |  |
| Post-Recession | 1 | 16 | 1148773.842 | 1148896.358 | - | - | - | - |
|  | 2 | 25 | 1113309.595 | 1113501.027 | 0.000 | 0.000 | 0.000 | 1.000 |
|  | 3 | 34 | 1091620.003 | 1091880.350 | 0.000 | 0.000 | 0.000 | 1.000 |
|  | 4 | 43 | 1074274.753 | 1074604.015 | 0.000 | 0.000 | 0.000 | 0.989 |
|  | 5 | 52 | 1062755.724 | 1063153.902 | 0.000 | 0.023 | 0.024 | 0.990 |
|  | 6 | 61 | 1048437.942 | 1048905.035 | 0.000 | 0.013 | 0.014 | 0.991 |
|  | 7 | 70 | 1037233.623 | 1037769.631 | 0.000 | 0.000 | 0.000 | 0.989 |
|  | 8 | 79 | 1030404.576 | 1031009.500 | 0.000 | 0.000 | 0.000 | 0.982 |
|  | 9 | 88 | 1024901.946 | 1025575.786 | 0.000 | 0.143 | 0.144 | 0.968 |

| **Table A2. Model Fit Statistics Used to Determine Class Enumeration for Male Respondents** | | | | | | | | |
| --- | --- | --- | --- | --- | --- | --- | --- | --- |
|  | **Classes** | **# Parameters** | **AIC** | **BIC** | **BLRT *p* value** | **VLMR *p* value** | **LMRT *p* value** | **Entropy** |
| Pre-Recession | 1 | 16 | 1255637.665 | 1255763.449 | - | - | - | - |
|  | 2 | 25 | 1196153.710 | 1196350.248 | 0.000 | 0.000 | 0.000 | 1.000 |
|  | 3 | 34 | 1170574.123 | 1170841.415 | 0.000 | 0.005 | 0.005 | 1.000 |
|  | 4 | 43 | 1145463.486 | 1145801.532 | 0.000 | 0.000 | 0.000 | 0.999 |
|  | 5 | 52 | 1125727.330 | 1126136.129 | 0.000 | 0.000 | 0.000 | 0.994 |
|  | 6 | 61 | 1107594.882 | 1108074.435 | 0.000 | 0.000 | 0.000 | 0.995 |
|  | 7 | 70 | 1072785.229 | 1073113.078 | 0.000 | 0.731 | 0.731 | 0.999 |
|  |  |  |  |  |  |  |  |  |
| Recession | 1 | 16 | 633793.954 | 633908.762 | - | - | - | - |
|  | 2 | 25 | 606301.799 | 606481.187 | 0.000 | 0.000 | 0.000 | 1.000 |
|  | 3 | 34 | 592839.578 | 593083.547 | 0.000 | 0.023 | 0.024 | 1.000 |
|  | 4 | 43 | 579616.985 | 579788.886 | 0.000 | 0.000 | 0.000 | 0.999 |
|  | 5 | 52 | 569941.092 | 570314.220 | 0.000 | 0.000 | 0.000 | 0.994 |
|  | 6 | 61 | 561031.500 | 561469.208 | 0.000 | 0.357 | 0.360 | 0.995 |
|  |  |  |  |  |  |  |  |  |
| Post-Recession | 1 | 16 | 957288.116 | 957409.425 | - | - | - | - |
|  | 2 | 25 | 914305.043 | 914494.589 | 0.000 | 0.000 | 0.000 | 1.000 |
|  | 3 | 34 | 894153.861 | 894411.644 | 0.000 | 0.002 | 0.002 | 1.000 |
|  | 4 | 43 | 875301.816 | 875627.835 | 0.000 | 0.000 | 0.000 | 0.999 |
|  | 5 | 52 | 860142.212 | 860536.467 | 0.000 | 0.000 | 0.000 | 0.994 |
|  | 6 | 61 | 845728.776 | 846191.268 | 0.000 | 0.000 | 0.000 | 0.995 |
|  | 7 | 70 | 834546.902 | 835077.630 | 0.000 | 0.000 | 0.000 | 0.994 |
|  | 8 | 79 | 811792.436 | 812391.401 | 0.000 | 0.720 | 0.720 | 0.994 |
